# Analysis of the thickness characteristics of the left atrial posterior wall and its correlation with the low and no voltage areas of the left atrial posterior wall in patients with atrial fibrillation

**DOI:** 10.1186/s13019-024-02658-2

**Published:** 2024-04-06

**Authors:** Longchang Li, Lijun Li, Dezhi Yang, Shuxiong Nong, Cheng Luo, Chun Gui

**Affiliations:** 1 Department of Cardiology, The First Affiliated Hospital of Guangxi Medical University, No. 6 Shuangyong Road, Nanning, 530021 Guangxi People’s Republic of China; 2 Guangxi Key Laboratory Base of Precision Medicine in Cardio-Cerebrovascular Diseases Control and Prevention, Nanning, 530021 Guangxi People’s Republic of China; 3 Guangxi Clinical Research Center for Cardio-Cerebrovascular Diseases, Nanning, 530021 Guangxi People’s Republic of China; 4 Department of Cardiology, The Fifth Affiliated Hospital of Guangxi Medical University, Nanning, 530022 Guangxi People’s Republic of China; 5 Department of Cardiology Second Ward, Hechi People’s Hospital, Hechi, 547000 Guangxi People’s Republic of China

**Keywords:** Atrial fibrillation, Left atrial posterior wall thickness, Left atrial posterior wall, Low-voltage zone, Voltage-free zone

## Abstract

**Objective:**

To analyze the relationship between the thickness of the left atrial posterior wall and the low and no voltage zones in the left atrial posterior wall in patients with atrial fibrillation (AF).

**Methods:**

61 patients admitted to our cardiology department for AF and radiofrequency ablation of AF from January 1, 2020 to May 30, 2022 were enrolled according to inclusion and exclusion criteria. The atrial wall thickness was measured by CT scan. Baseline data, preoperative cardiac ultrasound data, preoperative biochemical parameters, low voltage zone (fibrotic zone) and no voltage zone (scar zone) in the left atrial posterior wall area, and various parameters of posterior left atrial wall thickness were collected.

**Results:**

The differences of the thickness between the upper, middle and lower mean levels of the left atrial posterior wall were statistically significant (*P* = 0.004). The results showed that body mass index was weakly positively correlated with the mean level of total left atrial posterior wall thickness (*r* = 0.426, *P* = 0.001) and was statistically significant. The remaining indices were positively or negatively correlated with the mean level of total left atrial posterior wall thickness, but none were statistically significant (*P* > 0.05).

**Conclusions:**

Both left atrial posterior wall low-voltage zone and voltage-free zone were positively correlated with the mean total left atrial posterior wall thickness, and left atrial posterior wall low-voltage zone and voltage-free zone were significantly positively correlated. Body mass index was weakly positively correlated with total left atrial posterior wall thickness.

**Supplementary Information:**

The online version contains supplementary material available at 10.1186/s13019-024-02658-2.

## Introduction

Atrial fibrillation (AF) is a type of arrhythmia and a common cause of ischemic stroke [1]. Its incidence increases with age, particularly in individuals aged 80 years and older, where the prevalence ranges from 10 to 12% [2]. The pathological mechanisms of AF are complex and still under investigation. Transcatheter radiofrequency ablation (RFCA) is the preferred treatment method for AF, and macrocyclic pulmonary vein isolation (CPVI) not only eliminates the triggers within the pulmonary veins but also improves the structure of the pulmonary venous vestibule [3]. Furthermore, CPVI addresses the trigger foci outside the pulmonary veins and the ganglion plexus in the vestibular region, partially reversing left atrial remodeling. Recent studies have shown a strong association between low voltage in the left atrium, as detected by electroanatomical marker systems, and delayed gadolinium imaging in MRI [4]. Low voltage in the left atrium also increases the risk of AF recurrence, and supplementing pulmonary vein isolation with ablation of the low voltage zone improves sinus rhythm maintenance in AF patients. Previous research has demonstrated a significant link between the atrial low voltage zone (LVZ) and the progression of AF, as well as the development of atrial fibrosis, as measured by atrial bipolar potentials using a pressure scaler catheter [5]. During radiofrequency ablation of AF, the presence of the left atrial low voltage zone, detected using a pressure-calibrated measurement catheter, is associated with clinical risk factors.

In patients with AF, the left posterior atrial wall exhibits areas of low voltage and voltage-free zones, which correspond to left atrial fibrosis and scarring, respectively [6]. Previous reports have indicated that left atrial scarring is associated with the development of AF thrombosis and stroke [7]. Additionally, stroke patients have been found to have significantly more left atrial fibrosis [8]. These findings suggest that preventing the development of thrombosis and stroke in AF patients should focus on addressing left atrial fibrosis and scarring. AF can also lead to cardiac enlargement. The exact cause of AF is still unclear, but common factors include developmental malformations of the left atrium, right atrium, and medications, often resulting in arrhythmias and bradycardia. If left untreated, AF can lead to progressive myocardial expansion and eventually result in cardiomegaly [9]. Patients with AF often have a large left atrium and a thick posterior left atrial wall. There is a strong association between posterior left atrial wall thickness and the development of AF [10]. Left atrial posterior wall thickness is also considered a risk factor for AF [11]. However, studies investigating the correlation between left atrial posterior wall thickness and the low voltage region of the left atrial posterior wall in AF patients are limited. Therefore, this study aims to explore the relationship between clinical characteristics and left atrial posterior wall thickness in AF patients, as well as the association between the clinical characteristics of AF and the presence of low voltage zones and voltage-free zones in the left atrial posterior wall.

## Methods

### General information

A total of 61 patients admitted to The First Affiliated Hospital of Guangxi Medical University for AF with radiofrequency ablation of AF from January 1, 2020 to May 30, 2022 were enrolled according to the inclusion and exclusion criteria. The study was approved by The First Affiliated Hospital of Guangxi Medical University. Written informed consent was obtained from all individuals included in this study.

The study was reviewed by an ethics committee before it was conducted.

Inclusion criteria: (1) diagnosis of AF by our institution; (2) no previous history of AF ablation; (3) age of all subjects greater than or equal to 18 years; (4) no attached thrombus in the left atrium.

Exclusion criteria: (1) those who have had a myocardial infarction within 180 days; (2) history of coronary heart disease related medical or surgical procedures within 3 months; (3) those with atrioventricular block, liver or kidney injury, connective tissue or inflammatory disease, pulmonary heart disease, thyroid disease, pericardial disease, myocarditis, malignancy, etc.; (4) those who have had a stroke within 30 days; (5) those treated with corticosteroids, immunosuppressive and/or chemotherapy; (6) those with incomplete information.

### Collection of information

Patient data were collected from electronic medical records, including information such as the type of AF, gender, smoking status, hypertension, coronary artery disease, diabetes, combined atrial flutter, duration of AF, age, systolic and diastolic blood pressure, heart rate, body mass index, CHA2DS2-VASc score, and preoperative cardiac ultrasound indices. The cardiac ultrasound indices included measurements of anterior-posterior left atrial diameter, transverse left atrial diameter, end-diastolic left ventricular diameter, end-systolic left ventricular diameter, right ventricular diameter, ejection fraction (EF), cardiac output (CO), mitral regurgitation velocity, differential pressure, and instantaneous regurgitant flow. Additionally, preoperative biochemical parameters such as Pro-BNP, total cholesterol, high-density cholesterol, low-density cholesterol, triglycerides, creatinine, endogenous creatinine clearance, fasting blood glucose, fasting glucose, 2-hour postprandial blood glucose, glycated hemoglobin HbA1c, FT3, FT4, and TSH were collected. During the operation, data on the posterior wall low voltage zone, posterior wall low voltage zone as a percentage of the left atrium, posterior wall no voltage zone, posterior wall no voltage zone as a percentage of the left atrium, posterior wall area, and posterior wall area as a percentage of the left atrium were also collected. Indicators of posterior wall thickness of the left atrium, including overall mean, upper posterior wall mean, lower posterior wall mean, left posterior wall mean, right posterior wall mean, horizontal mid-mean, and vertical mid-mean, were measured.

### Observation indicators

#### Clinical characteristics, cardiac ultrasound indices and biochemical indices

The study observed the clinical characteristics, cardiac ultrasound indices, and biochemical indices of patients with AF. The aim was to analyze the correlation between the clinical characteristics of AF patients and the thickness of the left atrial posterior wall, as well as the correlation between the clinical characteristics of AF and the presence of low-voltage and voltage-free zones in the left atrial posterior wall. Additionally, the correlation between the thickness of the left atrial posterior wall and the low-voltage and voltage-free zones was examined.

### CT scan of left atrium

A 64-slice spiral CT scanner (GE, US) was used for the CT scan of the left atrium. The scan was performed in the supine position from the thoracic inlet to the upper edge of the diaphragm without ECG gating. The scan parameters included a voltage of 80 kVp, rotation speed of the X-ray tube of 0.33 s/cycle, tube current of 120 mA, collimator size of 64 × 0.625 mm, scan field of 250 mm × 250 mm, matrix size of 512 × 512, and pitch of 0.9:1. A nonionic contrast agent (uitravist or iopromide) was injected into the dorsal hand vein using a double high-pressure syringe (Stellant, Medrad, US) at a rate of 1–4 ml/s.

### Measurement of the thickness of the posterior wall of the left atrium

**Fig. 1:**
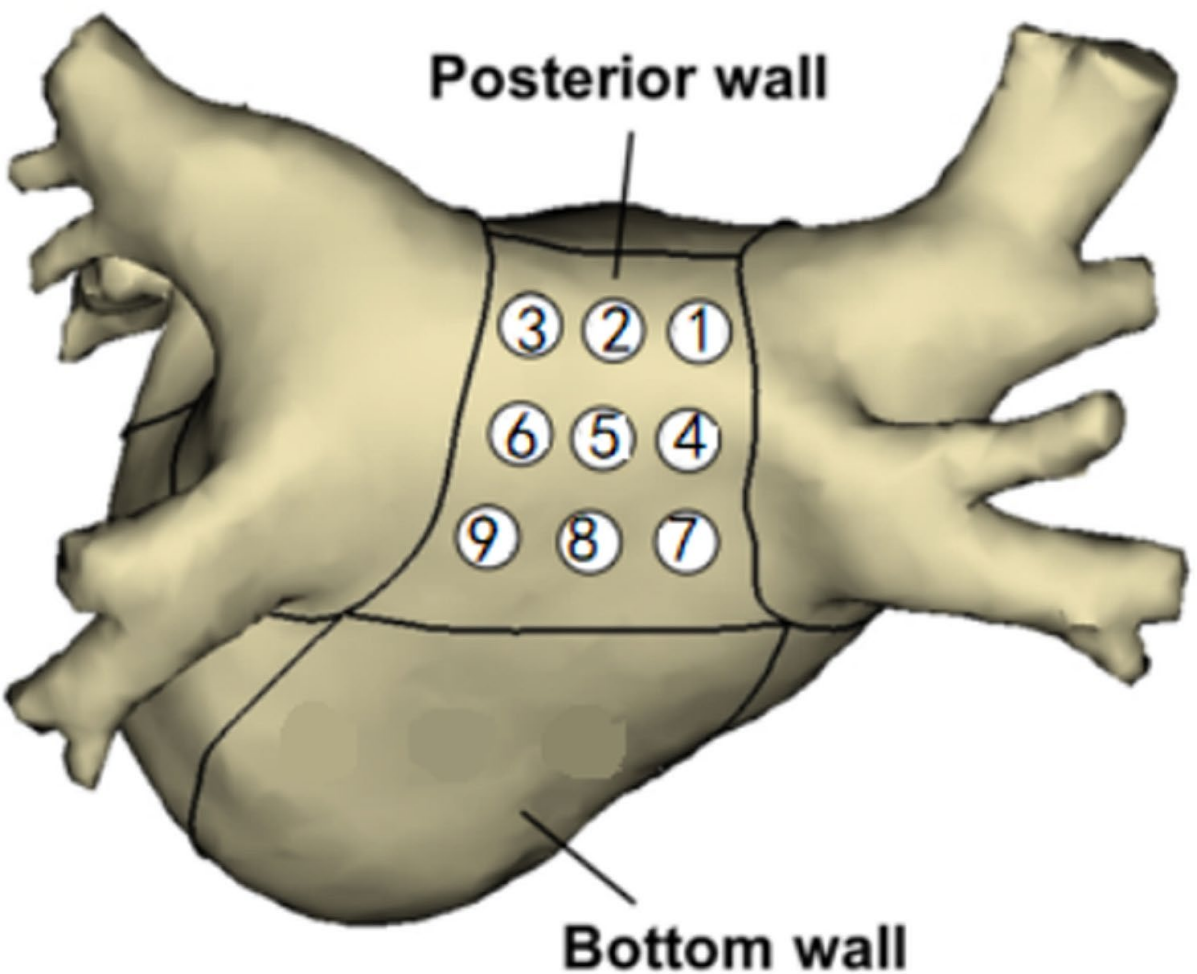
Measurement of the thickness of the posterior wall of the left atrium

**Fig. 2:**
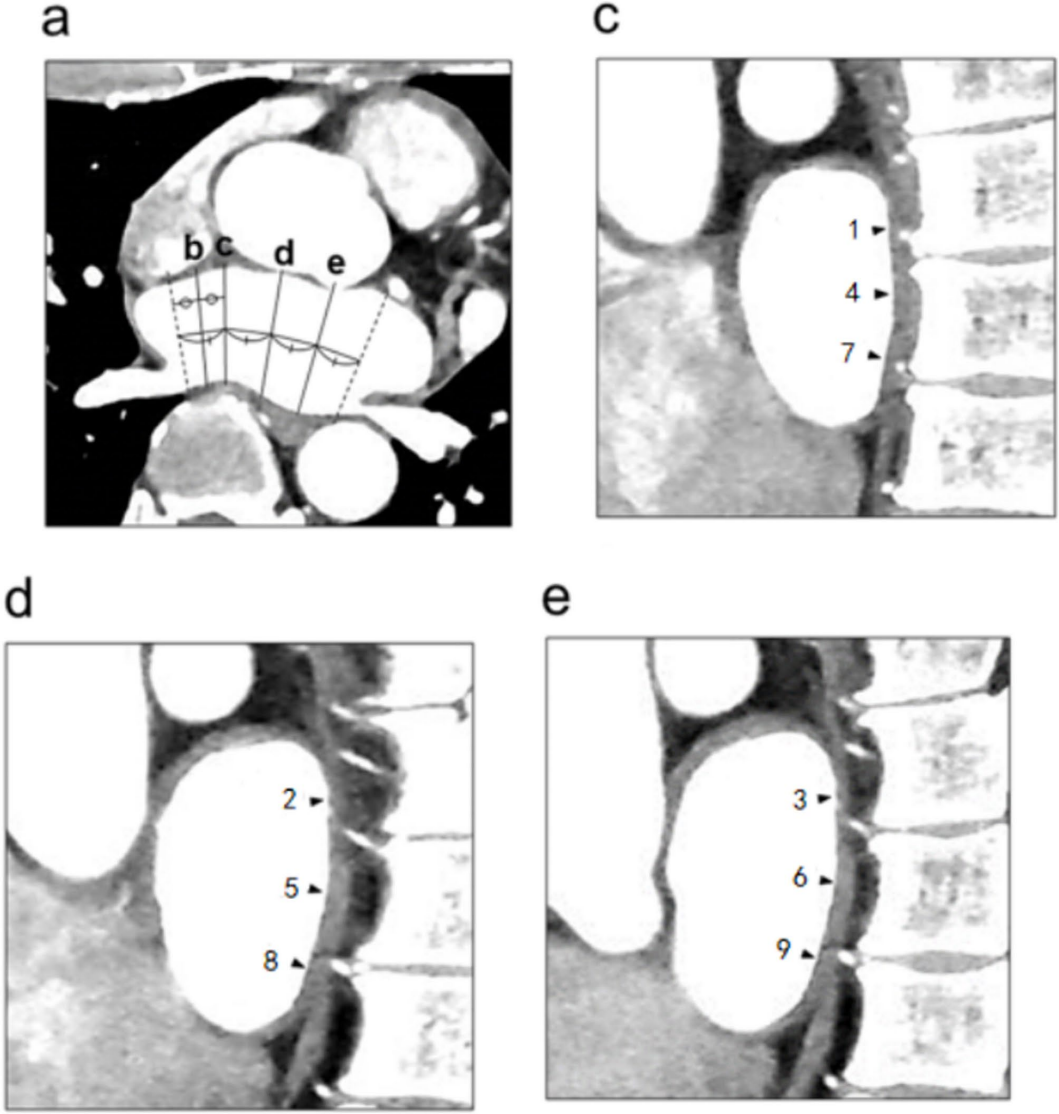
Left atrial measurement point diagram. **a**. In the oblique horizontal position for measuring the left atrial transverse diameter, the left atrial body between the bilateral pulmonary venous sinuses (dotted lines) is divided equally into four parts by lines **c**-**e**. Wall thickness is measured along these lines (**c**-**e**) in the oblique sagittal plane perpendicular to the transverse left atrial diameter. Figures **c**, **d** and **e** correspond to the lines in figure **a**. The arrows indicate the measurement points. Measurement point 4 is the midpoint of the posterior wall; measurement point 1 is located in the upper 1/3 of the line connecting measurement point 4 to the top line of the left room in figure **c**. Measurement points 2 and 3 are measured in the same way as measurement point 1; measurement point 7 is located in the lower 2/3 of the line connecting measurement point 4 to the bottom line of the left room in figure **c**. Measurement points 8 and 9 are measured in the same way as measurement point 7; the figures shown correspond to the positions of the measurement points shown in Fig. 1. Measurement point 1 = top right of the rear wall, measurement point 2 = top middle of the rear wall, measurement point 3 = top left of the rear wall, measurement point 4 = middle right of the rear wall, measurement point 5 = middle of the rear wall, measurement point 6 = middle left of the rear wall, measurement point 7 = bottom right of the rear wall, measurement point 8 = bottom middle of the rear wall, measurement point 9 = bottom left of the rear wall, comparison between top middle and bottom of the rear wall for 1, 2, 3; 4, 5, 6; 7, 8,9 The comparison between the three groups of data is 3, 6, 9; 2, 5, 8; 1, 4, 7 for the left-centre-right comparison of the rear wall

The thickness of the left atrial posterior wall was measured using cross-section reconstruction with a slice thickness of 0.625 mm. The reconstructed data were transferred to an image post-processing workstation (Advantage Workstation 4.3, GE) for analysis. Multi-planar reconstruction (MPR), maximum intensity projection (MIP), and volume rendering (VR) techniques were used to analyze the reconstructed left atrium and pulmonary vein. The thickness at each location was measured three times by an experienced CT technician, and the measurements were averaged. An independent examiner reviewed and double-checked these measurements. Fig 1-2 shows the measurement scheme of the posterior wall of the left atrium.

### Left atrial electroanatomical marker measurement (EAM) and catheter radiofrequency ablation

Left atrial bipolar voltage was measured using the Carto 3D electroanatomical calibration system and a PentaRay NAV eco High-Density Mapping Catheter (Johnson & Johnson, USA). Voltage measurements were taken after atrial septal puncture, with over 300 sampling points for each patient. The sampling time window was set to be greater than 2 s. Local voltages ranging from 0.1 to 0.5 mV were defined as low voltage areas, while voltages below 0.1 mV were defined as no voltage areas. The total area of the left atrium was derived from the software. The percentage of the low voltage zone area to the total left atrial area indicated the degree of fibrosis in the patient’s left atrium, and the percentage of the voltage-free zone area to the total left atrial area indicated the size of the atrial scar zone.

### Statistical analysis

SPSS 25.0 was used to analyse the measurement data and count data collected in this study. The measurement data were tested for normality, and data that conformed to a normal distribution were expressed as (± s), and comparisons between groups were made using the independent samples t-test. Data that did not conform to a normal distribution were expressed using the median (quartiles) and the Mann-Whitney U test was used for comparison between groups. Categorical counts were expressed as percentages, and unordered categorical data were compared between groups using the χ^2^ or Fisher exact test; ordered categorical data were compared between groups using the Mann-Whitney U test. Correlations were analysed by Spearman for non-normals and Pearson for normally distributed indicators; *P* < 0.05 was considered a statistically significant difference.

## Results

### General information on all patients

Baseline information on subjects’ AF type, gender, smoking, hypertension, coronary artery disease, diabetes, and combined atrial flutter is shown in Table 1. There were 34 males and 27 females. Totally, 36 patients were diagnosed with persistent AF, while 25 patients were diagnosed with paroxysmal AF. Twenty-four patients (39.34) were combined with atrial flutter. Patient clinical data, laboratory, echocardiographic parameters were shown in Supplemental Tables.

**Table 1:** Baseline information on patient classification

Indicators	Classification	N	%
Types of Atrial Fibrillation	Persistent	36	59.02
	Paroxysmal	25	40.98
Gender	Male	34	55.74
	Female	27	44.26
Smoking	No	48	78.69
	Yes	13	21.31
Arterial hypertension	No	33	54.10
	Yes	28	45.90
Coronary atherosclerosis	No	55	90.16
	Yes	6	9.84
Diabetes	No	54	88.52
	Yes	7	11.48
Atrial flutter	No	37	60.66
	Yes	24	39.34

### Indicators of low voltage zone (fibrotic zone) and no voltage zone (scar zone) in the posterior wall area of the left atrium

The indicators for the posterior wall area of the left atrium, the low voltage area of the posterior wall (fibrotic area) and the no voltage area (scarred area) are shown in Table 2. For the posterior wall low voltage area, the posterior wall low voltage area as a percentage of the left atrium, the posterior wall no voltage area, the posterior wall no voltage area as a percentage of the left atrium, the posterior wall area, and the posterior wall area as a percentage of the left atrium.

**Table 2:** Comparison of upper, middle and lower mean levels of left posterior atrial wall thickness

Indicators	Level	X^2^	P	Top vs. Bottom	Top vs.	Lower vs. Middle
Upper mean value (mm)	1.41 (1.3, 1.575)	11.031	0.004	0.009	0.518	0.069
Lower mean value (mm)	1.47 (1.33, 1.635)					
Mean value across the middle (mm)	1.45 (1.23, 1.595)					

### Thickness of the posterior wall of the left atrium

The overall mean (1.48 ± 0.21) mm, upper posterior wall mean [1.41 (1.30, 1.58)] mm, lower posterior wall mean [1.47 (1.33, 1.64)] mm, left posterior wall mean (1.57 ± 0.32) mm, right posterior wall mean [1.41 (1.26, 1.55)] mm, and horizontal mid mean [1.45(1.23, 1.60)] mm, and vertical mid-mean (1.5 ± 0.24) mm are shown in Table 3.

**Table 3:** Comparison of left, middle and right mean levels of left posterior atrial wall thickness

Indicators	Level	X2	P	Left vs. Right	Left vs. centre	Right vs.
Left mean (mm)	1.57 ± 0.32	22.366	< 0.001	< 0.001	0.039	0.001
Right mean (mm)	1.41 (1.26, 1.55)					
Mean vertical centre value (mm)	1.50 ± 0.24					

The differences of the thickness between the upper, middle and lower mean levels were statistically significant (*P* = 0.004), with the upper mean level being significantly lower than the lower mean level (*P* = 0.009), and the differences of the thickness between the upper and middle means and the lower and middle means were not statistically significant (*P* > 0.0125) (Table 2).

The differences between the left, middle and right mean levels were statistically significant (*P* < 0.0001 < 0.05), with the left mean level being significantly higher than the right mean level (*P* < 0.0001), the middle mean level being significantly higher than the right mean level (*P* = 0.001) and the difference between the left and middle means not being statistically significant (*P* = 0.039) (Table 3).

### Correlation analysis between clinical characteristics of AF and mean left atrial posterior wall thickness

The difference in mean left atrial posterior wall total thickness levels was statistically significant (*P* < 0.05) between the sexes, with men having a significantly greater mean left atrial total thickness than women. The difference in mean left atrial total posterior wall thickness between those with and without diabetes was statistically significant (*P* < 0.05), with those with diabetes having a greater mean left atrial posterior wall total thickness than those without diabetes. There was no statistically significant difference in the mean left atrial posterior wall total thickness levels between the different types of AF, smoking, hypertension, coronary artery disease and atrial flutter (*P* > 0.05) (Supplemental Tables).

Correlation analysis was performed between the continuity index and the mean level of total left atrial posterior wall thickness, where Spearman correlation was used for non-normals and Pearson correlation was used for normally distributed indexes. The results showed that body mass index was weakly positively correlated with the mean level of total left atrial posterior wall thickness (*r* = 0.426, *P* = 0.001) and was statistically significant. The rest of the indicators were positively or negatively correlated with the mean level of total left atrial posterior wall thickness, but none were statistically significant (*P* > 0.05) (Supplemental Tables).

### Correlation analysis of low-voltage zone and voltage-free zone with mean total left atrial posterior wall thickness

Both intraoperative low-voltage zone and voltage-free zone were weakly yet significantly correlated with the mean total left atrial posterior wall thickness (*P* < 0.05). Intraoperative low-voltage zone and voltage-free zone were significantly positively correlated (*r* = 0.609, *P* < 0.0001) and statistically significant (Supplemental Tables).

### Correlation analysis between clinical characteristics of patients with AF and areas of low-voltage zone and voltage-free zone of the left atrial posterior wall

The differences in the left atrial posterior wall low-voltage zone were statistically significant (*P* < 0.05) between the different types of AF, with persistent left atrial posterior wall low-voltage zone being significantly greater than paroxysmal, and no statistically significant differences in the left atrial posterior wall low-voltage zones of other clinical features of AF (Table 4).

**Table 4:** Comparison of different clinical features with areas of left atrial low-voltage zone

Indicators	Category	Number of examples	Left atrial low-voltage zone	Z	P
Types of Atrial Fibrillation	Persistent	36	4.65 (2.025, 7.65)	-5.213	< 0.001
	Paroxysmal	25	0.00 (0.00, 0.20)		
Gender	Male	34	1.65 (0, 5.225)	-0.734	0.463
	Female	27	2.00 (0.00, 7.50)		
Smoking	No	48	2.15 (0.00, 6.43)	-1.245	0.213
	Yes	13	0.80 (0.00, 3.40)		
Suffering from high blood pressure	No	33	2.20 (0.00, 7.05)	-1.053	0.292
	Yes	28	1.00 (0.00, 4.45)		
Suffering from coronary heart disease	No	55	2.00 (0.00, 5.30)	-0.025	0.980
	Yes	6	1.80 (0.00, 6.20)		
Diabetes	No	54	1.85 (0.00, 5.53)	-0.082	0.935
	Yes	7	2.00 (0.00, 4.40)		
With or without atrial flutter	None	37	2.20 (0.00, 5.30)	-0.518	0.604
	Yes	24	0.95 (0.00, 6.63)		

The difference in voltage-free zone size was statistically significant (*P* < 0.05) between the different types of AF, with persistent voltage-free zone size being significantly greater than paroxysmal, and no statistically significant difference in voltage-free zone size for other clinical features of AF (Table 5).

**Table 5:** Comparison of different clinical features with voltage-free zone size

Indicators	Category	Number of examples	voltage-free zone size	Z	P
Types of Atrial Fibrillation	Persistent	36	1.25 (0.00, 4.85)	-4.281	< 0.0001
	Paroxysmal	25	0.00 (0.00, 0.00)		
Gender	Male	34	0.00 (0.00, 1.40)	-1.524	0.128
	Female	27	0.60 (0.00, 4.00)		
Smoking	No	48	0.25 (0, 20.075)	-1.173	0.241
	Yes	13	0.00 (0.00, 0.70)		
Suffering from high blood pressure	No	33	0.40 (0.00, 1.95)	-1.002	0.316
	Yes	28	0.00(0, 10.675)		
Suffering from coronary heart disease	No	55	0.10 (0.00, 1.80)	-0.452	0.652
	Yes	6	0.00 (0.00, 3.75)		
Diabetes	No	54	0.05 (0.00, 1.85)	-0.06	0.952
	Yes	7	0.80 (0.00, 1.20)		
With or without atrial flutter	None	37	0.10 (0.00, 2.15)	-0.087	0.931
	Yes	24	0.05 (0.00, 1.775)		

Age, heart rate, anterior-posterior left atrial diameter, transverse left atrial diameter, mitral instantaneous regurgitant flow, and Pro-BNP were significantly and positively correlated with the posterior wall low-voltage zone (*R* > 0, *P* < 0.05), and heart rate, anterior-posterior left atrial diameter, transverse left atrial diameter, and Pro-BNP were significantly and weakly positively correlated with the left atrial posterior wall voltage-free zone (*R* > 0, *P* < 0.05) (Supplemental Tables).

## Discussion

The main pathophysiological features of AF include disturbances in ventricular rate, impaired cardiac function, and atrial appendage thrombosis [12]. The presence of a low-voltage, voltage-free zone in the left atrial posterior wall has a negative impact on AF and can contribute to thrombosis and recurrence of atrial arrhythmia after radiofrequency ablation [13]. The thickness of the posterior left atrial wall is an independent risk factor for paroxysmal AF. In this study, we collected clinical data from 61 AF patients and analyzed the relationship between left atrial posterior wall thickness and the presence of low and no voltage zones in the left atrial posterior wall.

The study revealed statistically significant differences in the upper, middle, and lower mean levels of left atrial posterior wall thickness. Specifically, the upper mean level was significantly lower than the lower mean level, and there were significant differences between the left, middle, and right mean levels. These findings indicate that the thickness of the left atrial posterior wall varies at different sites. A previous study [14] also demonstrated that the left atrial posterior wall thickness was not uniform in both AF and non-AF groups, with variations in the upper, middle, and lower mean levels. When comparing the left atrial posterior wall thickness with different clinical characteristics of AF, it was observed that the mean total left atrial posterior wall thickness was significantly greater in men than in women. Additionally, patients with diabetes had a greater mean total left atrial posterior wall thickness compared to those without diabetes, while minimal differences were observed in other indicators. A weak positive correlation, which was statistically significant, was found between body mass index and the mean level of total left atrial posterior wall thickness, as indicated by the continuity index. The remaining indicators showed positive or negative correlations with the mean level of total left atrial posterior wall thickness, but none of them reached statistical significance. These findings suggest that body mass index may be a factor influencing the total left atrial posterior wall thickness in AF.

The left atrial low voltage zone is present in approximately 1/5 to 1/4 of AF patients. Patients with persistent AF have a larger left atrial low voltage zone and area compared to those with paroxysmal AF. The low voltage zone is commonly found in the anterior left atrial wall, septum, and posterior left atrial wall [15]. Delayed gadolinium-enhanced cardiac magnetic resonance imaging (DE-cMRI) clearly reveals areas of myocardial scarring or fibrosis in the left atrium. The low voltage zone in the posterior left atrial wall (fibrosis) is strongly associated with the development and perpetuation of AF [16]. AF and left atrial fibrosis have a mutually reinforcing relationship, where the electrical disturbances of AF lead to structural changes in the atria, including left atrial fibrosis. In turn, AF exacerbates the electrical remodeling in the atria, perpetuating the condition [17]. Histopathological examination of human atria has shown that patients with AF have more fibrosis compared to individuals in sinus rhythm, and the extent of fibrosis is significantly related to the duration of AF. Atrial fibrosis plays a crucial role in the maintenance of AF, and as the scar load increases, the rate of AF recurrence also rises. Recurrence after AF ablation is also associated with a low voltage zone in the posterior left atrial wall [18]. Both fibrosis in the low voltage zone and scar areas in the no voltage zone contribute to the development and prognosis of AF [19]. Further analysis of the relationship between posterior left atrial wall thickness and the low-voltage and voltage-free areas of the posterior left atrial wall in AF patients demonstrated a positive and weak correlation between intraoperative fibrosis in the low-voltage area, scar areas in the voltage-free area, and the total thickness of the left atrial posterior wall. This correlation was statistically significant. Additionally, a statistically significant positive correlation was found between intraoperative fibrosis and scar area, consistent with the aforementioned findings. The presence of persistently severe fibrosis in the left atrium leads to structural abnormalities that further increase the risk of thromboembolic events in AF patients.

In terms of the impact of different clinical characteristics of AF on fibrosis in the low voltage zone and scar zone of the left atrial posterior wall, it was observed that the size of the fibrosis zone and scar zone in the left atrial posterior wall was significantly larger in persistent AF compared to paroxysmal AF. Therefore, special attention should be given to patients with persistent AF to prevent the development of left atrial fibrosis and scar zones, which can lead to adverse events such as thrombosis and recurrence of atrial arrhythmia. In addition, a correlation analysis was conducted to examine the relationship between AF clinical characteristics and fibrosis in the low voltage zone and no voltage zone of the left atrial posterior wall. Age, heart rate, anterior-posterior left atrial diameter, left atrial transverse diameter, instantaneous regurgitant flow, and Pro-BNP showed significant positive correlations with the fibrosis zone of the posterior wall, while heart rate, anterior-posterior left atrial diameter, left atrial transverse diameter, and Pro-BNP showed significant positive correlations with the no voltage zone of the posterior wall. These correlations were weakly positive, suggesting that changes in these indicators may predict the occurrence of low voltage zones and voltage-free zones in the posterior wall of the left atrium. Therefore, it is crucial to monitor these indicators in patients with AF in clinical practice to anticipate potential risk events and provide timely prevention and treatment [20].

Furthermore, a previous study investigated the increase in linear blood flow velocity and volume flow during the spreading of the wave after a long pause between ventricular contractions in patients with AF [21]. The study found that the longer the pause between ventricular contractions, the greater the increase in arterial kinetics parameters. Another study demonstrated a significant relationship between P-wave peak time in lead DII and paroxysmal atrial fibrillation among patients with acute ischemic stroke [22].

In summary, both intraoperative fibrosis and scar areas showed positive correlations with the total thickness of the left atrial posterior wall, and there was a significant positive correlation between intraoperative fibrosis and scar areas. Body mass index showed a weak positive correlation with the total left atrial thickness. Age, heart rate, anterior-posterior left atrial diameter, transverse left atrial diameter, instantaneous regurgitant flow, and Pro-BNP were significantly and positively correlated with the fibrosis area of the posterior wall, while heart rate, anterior-posterior left atrial diameter, transverse left atrial diameter, and Pro-BNP were significantly and positively correlated with the scar area of the posterior wall in persistent AF. However, this study had certain limitations, such as a relatively small sample size from a single center. It is recommended to expand the sample size for subsequent analysis and further develop predictive models for AF risk events. Additionally, this study was a single-arm study without a control group, and it did not analyze diastolic dysfunction and cardiomyopathy, which are important factors that could be considered in future studies.

### Electronic supplementary material

Below is the link to the electronic supplementary material.

Supplementary Material 1

Supplementary Material 2

Supplementary Material 3

Supplementary Material 4

Supplementary Material 5

Supplementary Material 6

Supplementary Material 7

## Data Availability

All data generated or analysed during this study are included in this. Further enquiries can be directed to the corresponding author.

## Abbreviations

RFCA: Transcatheter radiofrequency ablation
CPVI: Choice and macrocyclic pulmonary vein isolation
LVZ: Low voltage zone
MPR: Multi-planner reconstruction
MIP: Maximum intensity projection
VR: Volume rendering
DE-cMRI: Delayed gadolinium-enhanced cardiac magnetic resonance imaging
