# Supplementary material for: Analysis of the thickness characteristics of the left atrial posterior wall and its correlation with the low and no voltage areas of the left atrial posterior wall in patients with atrial fibrillation
Source: J Cardiothorac Surg. 2024 Apr 6;19:187. doi: 10.1186/s13019-024-02658-2 (PMC10998308; doi:10.1186/s13019-024-02658-2)
Supplement: Supplementary file 1 — Supplementary Material 1 [file 13019_2024_2658_MOESM1_ESM.doc]

**Supplemental table 1** General information on subjects.

| Indicators | Level |
| --- | --- |
| Duration of atrial fibrillation (m) | 24.00 (9.75, 45.00) |
| Age (years) | 59.77±10.05 |
| Systolic blood pressure (mmHg) | 126.48±22.25 |
| Diastolic blood pressure (mmHg) | 79.44±13.28 |
| Heart rate (beats/min) | 85.00 (72.50, 98.75) |
| Body mass index (kg/m^2^ ) | 24.39±3.74 |
| CHA2DS2-VASc score (points) | 2.00 (1.00, 3.00) |
| Anterior-posterior left atrial diameter (mm) | 40.69±7.03 |
| Left atrial transverse diameter (mm) | 46.42±6.21 |
| Left ventricular diastolic end diameter (mm) | 50.61±6.13 |
| Left ventricular systolic end diameter (mm) | 31.00 (29.00, 35.75) |
| Right inner chamber diameter (mm) | 20.00 (19.00, 21.00) |
| EF (%) | 66.00 (60.25, 69.00) |
| CO (L/min) | 5.95±1.57 |
| Mitral regurgitation velocity (m/s) | 4.05±1.33 |
| Mitral differential pressure (HHmg) | 74.64±30.64 |
| Mitral instantaneous backflow (m^3^ /s) | 4.00 (3.00, 10.50) |
| Pro-BNP (pg/ml) | 636.50 (256.40, 967.90) |
| Total cholesterol (mmol/L) | 4.43±0.93 |
| High-density cholesterol (mmol/L) | 1.07 (0.94, 1.18) |
| Low density cholesterol (mmol/L) | 2.64±0.75 |
| Triglycerides (mmol/L) | 1.16 (0.88, 1.62) |
| Creatinine (umol/L) | 78.00 (63.00, 86.00) |
| Endogenous creatinine clearance (ml/min) | 76.06±18.14 |
| Fasting blood glucose (mmol/L) | 4.68 (4.46, 5.13) |
| Blood glucose (mmol/L) 2 hours postprandial | 6.20 (5.20, 8.36) |
| Glycated haemoglobin HbA1c (%) | 5.90 (5.70, 6.40) |
| FT3 (pmol/L) | 4.19 (4.05, 5.27) |
| FT4 (pmol/L) | 10.56±1.55 |
| TSH (uIU/L) | 1.96 (1.35, 3.16) |
