# Supplementary material for: Analysis of the thickness characteristics of the left atrial posterior wall and its correlation with the low and no voltage areas of the left atrial posterior wall in patients with atrial fibrillation
Source: J Cardiothorac Surg. 2024 Apr 6;19:187. doi: 10.1186/s13019-024-02658-2 (PMC10998308; doi:10.1186/s13019-024-02658-2)
Supplement: Supplementary file 2 — Supplementary Material 2 [file 13019_2024_2658_MOESM2_ESM.doc]

**Supplemental table 2** Indicators of the low voltage zone (fibrotic zone) and the no voltage zone (scar zone) in the posterior wall area of the left atrium.

| Indicators | Level |
| --- | --- |
| Low voltage area on the back wall (cm^2^) | 2.00 (0.00, 5.30) |
| Percentage of low voltage area on the back wall (%) | 1.00 (0.00, 2.53) |
| No voltage zone on the rear wall (cm^2^) | 0.10 (0.00, 1.80) |
| Percentage of no voltage zone on the rear wall (%) | 0.10 (0.00, 0.78) |
| Area of rear wall (cm^2^) | 12.70 (9.70, 15.70) |
| Percentage of area of rear wall (%) | 5.70 (4.63, 7.10) |
