# Supplementary material for: Analysis of the thickness characteristics of the left atrial posterior wall and its correlation with the low and no voltage areas of the left atrial posterior wall in patients with atrial fibrillation
Source: J Cardiothorac Surg. 2024 Apr 6;19:187. doi: 10.1186/s13019-024-02658-2 (PMC10998308; doi:10.1186/s13019-024-02658-2)
Supplement: Supplementary file 3 — Supplementary Material 3 [file 13019_2024_2658_MOESM3_ESM.doc]

**Supplemental table 3** Posterior wall thickness of the left atrium.

| Indicators | Level |
| --- | --- |
| Overall mean value (mm) | 1.48±0.21 |
| Mean value of upper rear wall (mm) | 1.41 (1.30, 1.58) |
| Mean value of lower rear wall (mm) | 1.47 (1.33, 1.64) |
| Average value of the left side of the rear wall (mm) | 1.57±0.32 |
| Mean value of right side of rear wall (mm) | 1.41 (1.26, 1.55) |
| Mean value across the middle (mm) | 1.45 (1.23, 1.60) |
| Mean vertical centre value (mm) | 1.5±0.24 |
