# Supplementary material for: Analysis of the thickness characteristics of the left atrial posterior wall and its correlation with the low and no voltage areas of the left atrial posterior wall in patients with atrial fibrillation
Source: J Cardiothorac Surg. 2024 Apr 6;19:187. doi: 10.1186/s13019-024-02658-2 (PMC10998308; doi:10.1186/s13019-024-02658-2)
Supplement: Supplementary file 4 — Supplementary Material 4 [file 13019_2024_2658_MOESM4_ESM.doc]

**Supplemental table 4** Comparison of different clinical characteristics with mean total left atrial posterior wall thickness.

| Indicators | Category | Number of examples | Average total left atrial posterior wall thickness | t | P |
| --- | --- | --- | --- | --- | --- |
| Types of Atrial Fibrillation | Persistent | 36 | 1.50±0.22 | 0.863 | 0.392 |
|  | Paroxysmal | 25 | 1.46±0.20 |  |  |
| Gender | Male | 34 | 1.54±0.21 | -2.535 | 0.014 |
|  | Female | 27 | 1.41±0.19 |  |  |
| Smoking | No | 48 | 1.447±0.21 | -1.189 | 0.239 |
|  | Yes | 13 | 1.55±0.19 |  |  |
| Suffering from high blood pressure | No | 33 | 1.46±0.23 | -1.068 | 0.290 |
|  | Yes | 28 | 1.52±0.18 |  |  |
| Suffering from coronary heart disease | No | 55 | 1.48±0.21 | -0.518 | 0.606 |
|  | Yes | 6 | 1.53±0.21 |  |  |
| Diabetes | No | 54 | 1.45±0.19 | -3.408 | 0.001 |
|  | Yes | 7 | 1.72±0.21 |  |  |
| With or without atrial flutter | None | 37 | 1.49±0.20 | 0.052 | 0.958 |
|  | Yes | 24 | 1.48±0.23 |  |  |
