# Supplementary material for: Analysis of the thickness characteristics of the left atrial posterior wall and its correlation with the low and no voltage areas of the left atrial posterior wall in patients with atrial fibrillation
Source: J Cardiothorac Surg. 2024 Apr 6;19:187. doi: 10.1186/s13019-024-02658-2 (PMC10998308; doi:10.1186/s13019-024-02658-2)
Supplement: Supplementary file 5 — Supplementary Material 5 [file 13019_2024_2658_MOESM5_ESM.doc]

**Supplemental table 5** Correlation analysis between clinical characteristics and mean total left atrial posterior wall thickness.

| Methods | Indicators | Correlation coefficient | P |
| --- | --- | --- | --- |
| Pearson Correlation | Age (years) | 0.006 | 0.961 |
|  | Systolic blood pressure (mmHg) | 0.055 | 0.675 |
|  | Diastolic blood pressure (mmHg) | 0.024 | 0.854 |
|  | Body mass index (kg/m^2^ ) | 0.426** | 0.001 |
|  | Anterior-posterior left atrial diameter (mm) | 0.207 | 0.109 |
|  | Left atrial transverse diameter (mm) | 0.097 | 0.496 |
|  | Left ventricular diastolic end-diameter (mm) | 0.090 | 0.492 |
|  | CO (L/min) | 0.114 | 0.388 |
|  | Endogenous creatinine clearance (ml/min) | -0.062 | 0.641 |
|  | FT4 (pmol/L) | 0.087 | 0.516 |
| Spielmann Rho | Duration of atrial fibrillation (h) | -0.243 | 0.061 |
|  | Heart rate (beats/min) | -0.037 | 0.776 |
|  | CHA2DS2-VASc score (points) | 0.058 | 0.654 |
|  | Left ventricular systolic end diameter (mm) | 0.124 | 0.342 |
|  | EF | 0.045 | 0.729 |
|  | Mitral instantaneous backflow (m^3^ /s) | 0.075 | 0.586 |
|  | Pro-BNP (pg/ml) | 0.174 | 0.223 |
|  | Glycated haemoglobin HbA1c (%) | 0.101 | 0.467 |
|  | FT3 (pmol/L) | -0.162 | 0.224 |
|  | TSH (uIU/L) | 0.124 | 0.356 |
