# Supplementary material for: Analysis of the thickness characteristics of the left atrial posterior wall and its correlation with the low and no voltage areas of the left atrial posterior wall in patients with atrial fibrillation
Source: J Cardiothorac Surg. 2024 Apr 6;19:187. doi: 10.1186/s13019-024-02658-2 (PMC10998308; doi:10.1186/s13019-024-02658-2)
Supplement: Supplementary file 6 — Supplementary Material 6 [file 13019_2024_2658_MOESM6_ESM.doc]

**Supplemental table 6** Correlation analysis of intraoperative low-voltage zone and voltage-free zone with mean total left atrial posterior wall thickness.

|  |  | low-voltage zone of the posterior wall | Posterior wall voltage-free zone | Overall mean |
| --- | --- | --- | --- | --- |
| low-voltage zone of the posterior wall | Correlation coefficient | 1 | 0.615** | 0.267 |
|  | Significance (two-tailed) | . | <0.001 | 0.037 |
|  | Number of cases | 61 | 61 | 61 |
| Posterior wall voltage-free zone | Correlation coefficient | 0.615** | 1 | 0.327* |
|  | Significance (two-tailed) | <0.001 | . | 0.010 |
|  | Number of cases | 61 | 61 | 61 |
| Overall mean | Correlation coefficient | 0.267* | 0.327* | 1 |
|  | Significance (two-tailed) | 0.037 | 0.0 | . |
|  | Number of cases | 61 | 61 | 61 |

Note: ** indicates a correlation (two side), P<0.01.

* indicates a correlation (two side), P<0.05
