# Supplementary material for: Analysis of the thickness characteristics of the left atrial posterior wall and its correlation with the low and no voltage areas of the left atrial posterior wall in patients with atrial fibrillation
Source: J Cardiothorac Surg. 2024 Apr 6;19:187. doi: 10.1186/s13019-024-02658-2 (PMC10998308; doi:10.1186/s13019-024-02658-2)
Supplement: Supplementary file 7 — Supplementary Material 7 [file 13019_2024_2658_MOESM7_ESM.doc]

**Supplemental table 7** Correlation analysis of clinical features with the low-voltage zone of the posterior wall of the left atrium and the voltage-free zone of the posterior wall.

| Indicators | low-voltage zone of the posterior wall | | Posterior wall voltage-free zone | |
| --- | --- | --- | --- | --- |
|  | Correlation coefficient | P | Correlation coefficient | P |
| Duration of atrial fibrillation (h) | 0.038 | 0.771 | -0.130 | 0.322 |
| Age (years) | 0.303* | 0.018 | 0.131 | 0.314 |
| Systolic blood pressure (mmHg) | -0.072 | 0.582 | -0.108 | 0.409 |
| Diastolic blood pressure (mmHg) | 0.080 | 0.540 | -0.099 | 0.447 |
| Heart rate (beats/min) | 0.397** | 0.002 | 0.279* | 0.029 |
| Body mass index (kg/m^2^ ) | -0.015 | 0.909 | -0.145 | 0.266 |
| CHA2DS2-VASc score (points) | 0.226 | 0.080 | 0.241 | 0.061 |
| Anterior-posterior left atrial diameter (mm) | 0.308* | 0.016 | 0.254* | 0.049 |
| Left atrial transverse diameter (mm) | 0.400** | 0.003 | 0.322* | 0.020 |
| Left ventricular diastolic end-diameter (mm) | -0.171 | 0.186 | -0.091 | 0.486 |
| Left ventricular systolic end diameter (mm) | -0.058 | 0.657 | 0.030 | 0.819 |
| EF | -0.094 | 0.471 | -0.226 | 0.080 |
| CO (L/min) | -0.032 | 0.808 | -0.121 | 0.355 |
| Mitral instantaneous backflow (m^3^ /s) | 0.361** | 0.007 | 0.237 | 0.082 |
| Pro-BNP (pg/ml) | 0.531** | <0.0001 | 0.426** | 0.002 |
| Endogenous creatinine clearance (ml/min) | -0.101 | 0.444 | 0.083 | 0.527 |
| Glycated haemoglobin HbA1c (%) | 0.072 | 0.603 | 0.105 | 0.452 |
| FT3 (pmol/L) | -0.093 | 0.486 | -0.254 | 0.054 |
| FT4 (pmol/L) | -0.043 | 0.751 | 0.064 | 0.635 |
| TSH (uIU/L) | 0.008 | 0.952 | 0.138 | 0.303 |

Note: * indicates correlation, P<0.05, ** indicates P<0.01.
